# A fully automatic system to assess foot collapse on lateral weight-bearing foot radiographs: A pilot study

**DOI:** 10.1016/j.cmpb.2021.106507

**Published:** 2022-01

**Authors:** J. Lauder, J. Harris, B. Layton, P. Heire, A. Sorani, M. DeSancha, A.K. Davison, C. Sammut-Powell, C. Lindner

**Affiliations:** a Salford Royal NHS Foundation Trust, United Kingdom; b East Lancashire Hospitals NHS Trust, Royal Blackburn Teaching Hospital, United Kingdom; c Division of Informatics, Imaging and Data Sciences, The University of Manchester, United Kingdom; d Division of Musculoskeletal and Dermatological Sciences, The University of Manchester, United Kingdom

**Keywords:** Clinical decision support system, Radiomics, Landmark localization, Charcot foot, Diabetes

## Abstract

**Background:**

Foot collapse is primarily diagnosed and monitored using lateral weight-bearing foot x-ray images. There are several well-validated measurements which aid assessment. However, these are subject to inter- and intra-user variability.

**Objective:**

To develop and validate a software system for the fully automatic assessment of radiographic changes associated with foot collapse; automatically generating measurements for calcaneal tilt, cuboid height and Meary's angle.

**Methods:**

This retrospective study was approved by the Health Research Authority (IRAS 244852). The system was developed using lateral weight-bearing foot x-ray images, and evaluated against manual measurements from five clinical experts. The system has two main components: (i) a Random Forest-based point-finder to outline the bones of interest; and (ii) a geometry-calculator to generate the measurements based on the point positions from the point-finder. The performance of the point-finder was assessed using the point-to-point error (i.e. the mean absolute distance between each found point and the equivalent ground truth point, averaged over all points per image). For assessing the performance of the geometry-calculator, linear mixed models were fitted to estimate clinical inter-observer agreement and to compare the performance of the software system to that of the clinical experts.

**Results:**

A total of 200 images were collected from 79 subjects (mean age: 56.4 years ±12.9 SD, 30/49 females/males). There was good agreement among all clinical experts with intraclass correlation estimates between 0.78 and 0.86. The point-finder achieved a median point-to-point error of 2.2 mm. There was no significant difference between the clinical and automatically generated measurements using the point-finder points, suggesting that the fully automatically obtained measurements are in agreement with the manually obtained measurements.

**Conclusions:**

The proposed system can be used to support and automate radiographic image assessment for diagnosing and managing foot collapse, saving clinician time, and improving patient outcomes.

## Highlights

• Clinical decision support system to automatically assess radiographic changes in foot collapse.
• Fully automatic generation of Meary's angle, calcaneal tilt, and cuboid height.
• Good manual measurement agreement among clinical experts for all three measurements.
• Automatically generated measurements in agreement with clinical expert measurements.
• First software system to automatically generate measurements in acquired foot collapse.

## 1 Introduction

Foot collapse is a frequently encountered problem in the developed world and is most commonly the result of Charcot neuroarthropathy, rheumatological conditions, or developmental foot anomalies [1]. In the context of Charcot neuroarthropathy, foot collapse is known to have a close association with ulceration, reduced mobility, and amputation. The progression of foot collapse is characterised by subluxation and fragmentation of bony foot segments, resulting in progressive mal-alignment [2]. Mal-alignments require early detection and quantification to guide management and prevent progression to deformity [3]. Deformity as a result of Charcot neuroarthropathy carries a 12-fold risk of amputation relative to diabetic individuals without Charcot neuroarthropathy [4]. Periodic radiographs are crucial in the diagnosis, monitoring and decision making [3,5,6]. Foot collapse is well demonstrated on a lateral weight-bearing foot radiograph, and a range of radiographic measures are used to describe the changes in the alignment of the longitudinal arch. Calcaneal tilt (aka calcaneal pitch), cuboid height, and Meary's angle (aka talus-first metatarsal angle) are the most commonly used measurements in clinical practice. These measures have been found to have relatively good reproducibility [3], to be distinctive for disease monitoring [7], and to be prognostic for ulcer genesis [8,9]. These measures are also widely used in surgical decision-making in cases of reconstructive foot surgery, with certain values warranting surgical reconstruction [10,11], and to determine surgical success and/or failure [12,13]. A disease classification scheme based on radiographic angles is widely used in orthopaedic foot and ankle decision-making to describe foot collapse subtypes [1].

In the experience of the authors, these measurements are underutilised in clinical practice due to the time-consuming nature of acquiring the measurements manually and the lack of robust inter- and intra-user reliability. It is hoped that an automated system may increase the application of these measurements and provide consistent results for the purposes for diagnosing and/or monitoring progression of foot collapse.

*Contributions:* We present a software system for the fully automatic assessment of radiographic changes associated with foot collapse. To the best of our knowledge, this is the first system to automate the radiographic assessment in diagnosing and monitoring acquired foot collapse. We performed a comprehensive evaluation of the system based on manual ground truth measurements from five clinical experts. The proposed system will save clinicians' time and improve patient outcomes via quantifying signs of disease and introducing assessment consistency.

## 2 Materials and methods

Approval for this retrospective study was obtained from the Health Research Authority (IRAS 244852). All data were anonymised and no patient informed consent was required.

### 2.1 Data collection

**Fig. 1:**
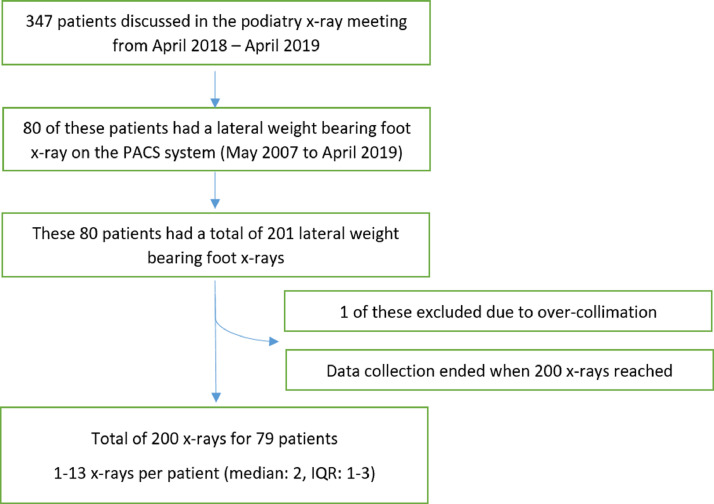
Flow diagram showing the number of included participants and corresponding number of x-ray images (IQR = interquartile range).

All patients were recruited retrospectively from the podiatry clinic radiology Multi-Disciplinary Team (MDT) case list at Salford Royal NHS Foundation Trust between April 2018 and April 2019 (Fig. 1). Every patient discussed in this MDT was eligible for recruitment, providing they had had a lateral weight-bearing foot radiograph as part of their podiatry assessment at any time from the initiation of the picture archiving and communication service (PACS) at the Trust in May 2007 to April 2019.

### 2.2 Manual measurements

**Fig. 2:**
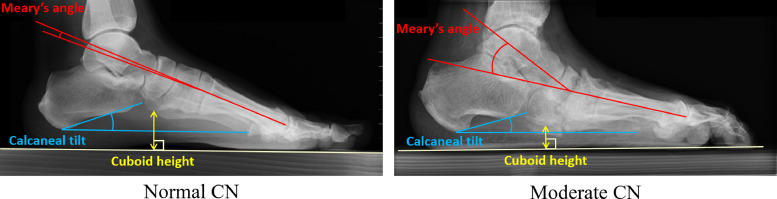
Visualisation of the measurements used in this study: calcaneal tilt (blue), cuboid height (yellow) and Meary's angle (red); (CN = Charcot neuroarthropathy).

Five clinical experts (three musculoskeletal consultants and two post-FRCR registrar radiologists) manually measured calcaneal tilt (in degrees), cuboid height (in mm) and Meary's angle (in degrees) for each of the images. Fig. 2 visualises the three measurements.

All manual measurements were taken on the GE PACS system at the Trust. Images where at least one of the radiologists was not able to take at least one of the measurements due to poor visibility were excluded from any of the geometric analyses. Even though the dataset includes several images per subject in some cases, all images were measured independently, and no link was made between different time points of the same subject.

**Fig. 3:**
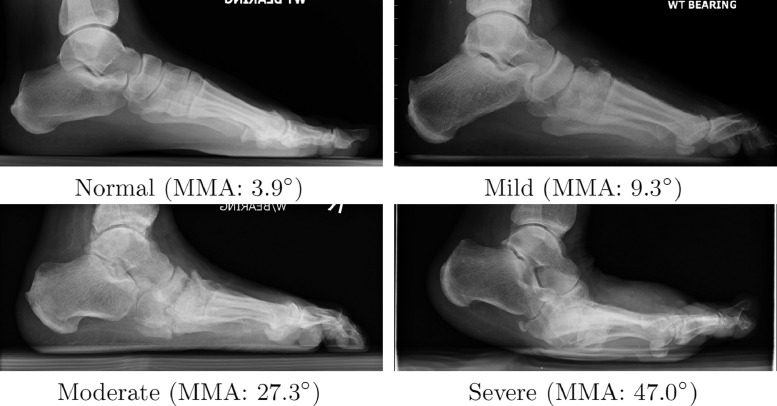
Examples of varying degrees of foot collapse based on Meary's angle (MMA = mean Meary's angle over all five clinical experts). The same images are classified as *normal, mild, severe* and *severe* based on clinical review. All radiographs have been cropped for better visualisation.

To evaluate how severity of disease affects the performance of our system, we classified the data based on Meary's angle. We applied the following commonly used thresholds to the mean value over all five observers: ≤ 4° *normal*, 4° < 15° *mild*, 15° ≤ 30° *moderate*, and 30° < *severe* (see Fig. 3 for examples of the different severity classes). Further, we had all images manually classified as *normal, mild* or *severe* based on clinical review by one of the clinical experts.

### 2.3 Fully automatic measurement system

The proposed software system has two main components: (i) a point-finder to outline the bones of interest; and (ii) a geometry-calculator to obtain the measurements based on the point positions from the point-finder.

**Fig. 4:**
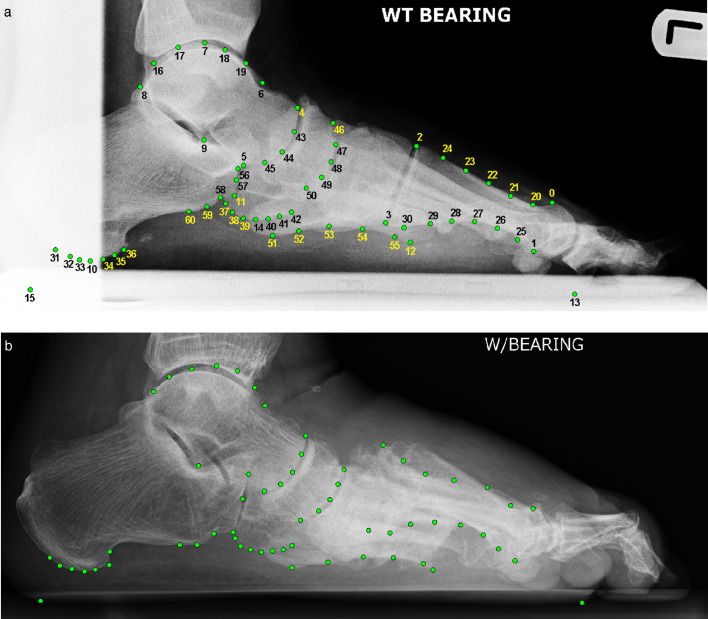
Annotation examples with 61 points (manually placed): (a) showing the 61 point positions and point indices for a *mild* case based on Meary's angle (MMA: 11.0°) with poor collimation. (b) showing the 61 point positions for a *mild* case based on Meary's angle (MMA: 6.3°) which shows advanced midfoot collapse. In this case, the 1st metatarsal has subluxed superiorly causing Meary's angle to be almost normal. This is a *severe* case based on clinical review. All radiographs have been cropped for better visualisation (MMA = mean Meary's angle over all five clinical experts).

*Point-finder:* The point-finder is based on Random Forest regression-voting Constrained Local Models (RFRV-CLM) [14], a machine learning method to accurately and robustly outline skeletal structures in radiographic images. It can easily be run on any computer without specific technical requirements, which is beneficial when aiming for the clinical integration of such a system. Full details on how to train the fully automatic point-finder are given in [14,15], here we describe our experimental set-up. We trained our point-finder to locate 61 points as shown in Fig. 4. The points were chosen such that they outline the bones of interest in studying foot collapse while including the minimal set of points required to calculate measurements for calcaneal tilt, cuboid height and Meary's angle. To provide the ground truth for training the point-finder system, all images were manually annotated with the 61 points by one of the clinical experts.

Based on the manual ground truth annotations, we ran 10-fold cross-validation (CV) experiments to generate fully automatic point-finder results for each image. CV experiments are systematic leave-some-out experiments, allowing to maximise the number of images used for training and validation while ensuring that the system is developed (trained) and assessed (tested) using different data. The cross-validation folds were defined randomly, taking into account that (i) multiple images of the same subject were included in the very same fold, and (ii) each fold included a balanced number of images and subjects. We included all images for the development of the point-finder.

For comparison, using the same data we also trained a SpatialConfiguration-Net (SCN) [16] to automatically locate the 61 points. SCN is a deep convolutional neural network (CNN) that was designed for landmark localisation in medical image data. We used the SCN implementation from the ‘MedicalDataAugmentationTool’ [16] (available at: https://github.com/christianpayer/MedicalDataAugmentationTool). The only changes made to this code were adjusting the number of landmarks, using the ‘DICOM’ image format, and changing the default number of folds from 3 to 10.

Both machine learning methods apply data augmentation techniques during training as described in [14,15] for the RFRV-CLM based point-finder and in [16] for the SCN.

*Geometry-calculator:* We used the point locations identified by the point-finder to automatically calculate calcaneal tilt, cuboid height and Meary's angle (all point indices refer to the indices as shown in Fig. 4a):

• *Calcaneal tilt (CT)*: The angle between (a) a line from point 10 to 11, and (b) a line from point 10 to 12.
• *Cuboid height (CH)*: The minimum distance between (a) a line from point 13 to 15, and (b) a piecewise cubic spline fitted to points 37-38-39-14-40-41-42.
• *Meary's angle (MA)*: The angle between (a) a centre line, metatarsal axis, between piecewise cubic splines fitted to points 20–24 and 25–28, respectively, and (b) a line through the centres of a line from point 4 to 5 and of a line from point 9 to the most superior point of a piecewise cubic spline fitted to points 8-16-17-7-18-19-6.

All geometric calculations were made using MATLAB R2017a.

### 2.4 Statistical analysis

To assess the performance of the point-finder, we report the point-to-point error (i.e. the mean absolute distance between each point resulting from applying the point-finder and the equivalent manual ground truth annotation point, averaged over all points per image), and the point-to-curve error (i.e. the mean absolute distance between each point resulting from applying the point-finder and a curve fitted to the manual ground truth annotation points, averaged over all points per image).

To assess the manual inter-observer agreement for each of the three geometric measurements, we report the intraclass correlation coefficient type 2 (ICC2) which is based on a two-way random effects model, considering both the images and observers as random effects. We also report the variance between observers (IOV), variance between images (IIV), and the proportion of variance explained by the differences between observers (POV). The latter was obtained by fitting a linear mixed-effects model to all manual geometric measurements with both the images and observers as random effects.

To assess the performance of the geometry-calculator in deriving the geometric measurements, we fitted a linear mixed-effects model with both the images and observers as random effects. The outcome was the difference between the *derived* geometric measurements (i.e. calculated using the points-based definitions above) and the manual ground truth geometric measurements provided by the observers. We report the results for the derived measurements based on both (i) the point positions automatically obtained from the point-finder, and (ii) the manual ground truth point positions. The latter is useful to evaluate how well the geometry-calculator reflects the geometric measurements, irrespectively of the point-finder performance for locating the points used to calculate the geometric measurements. We also calculated the Pearson correlation coefficient (PCC) to measure the association between the derived geometric measurements and the manual geometric measurements, determining the confidence intervals by bootstrapping with 1000 repeats. Further, we report the percentage of derived geometric measurements that are within observer range.

All statistical calculations were made using R v3.5.1 and RStudio v1.1.463.

## 3 Results

### 3.1 Data characteristics

A total of 347 patients were identified from the MDT meetings but only 80 of these had at least one image. For these patients, we exported 201 radiographs (including any historic foot radiographs if available on the PACS). However, one image had to be excluded due to poor collimation obscuring vital parts of the x-ray image (Fig. 1).

The final cohort consisted of 200 radiographs (73/127 females/males) taken from a total of 79 subjects (30/49 females/males). The mean age at image acquisition was 56.4 years ±12.9 SD. For every subject we had between 1 and 13 images (median: 2, interquartile range: 1–3). Multiple radiographs for the same subject were taken at different time points as part of their routine clinical care. The majority of referrals were for diabetic patients with an acute, atraumatic, hot foot with suspected Charcot neuroarthropathy. A small number of cases were post-traumatic cases with suspicion of foot collapse. The collected images represent a wide variety of progression of foot collapse; see Fig. 3 for some examples.

All radiographs were available in ‘DICOM’ image format. The pixel size varied across images. We used the pixel-spacing information from the DICOM header for each image to convert the measurements to mm.

### 3.2 Manual measurements

We excluded 12 images from all geometric analyses as for these images at least one of the radiologists was not able to take at least one of the measurements. Across all 188 images, the manually measured mean calcaneal tilt was found to be between 15.8° and 19.7°, the manually measured mean cuboid height between 18.5 mm and 22.5 mm, and the manually measured mean Meary's angle between 6.2° and 13.0° when measured by the clinical experts (Table 1).

**Table 1:** Means and standard deviations of calcaneal tilt (°), cuboid height (mm) and Meary's angle (°) measured across all 188 images by each of the five clinical experts (*E* = expert; SD = standard deviation).

	E1	E2	E3	E4	E5
**Measurement**	Mean ± SD				
**Calcaneal tilt**	19.0 ± 7.0	18.7 ± 6.6	19.7 ± 6.7	17.4 ± 7.0	15.8 ± 5.2
**Cuboid height**	18.5 ± 6.0	21.7 ± 6.4	21.4 ± 5.7	22.5 ± 6.6	18.5 ± 5.7
**Meary's angle**	8.2 ± 12.3	6.2 ± 13.7	13.0 ± 12.6	10.3 ± 14.2	10.7 ± 15.6

There was good agreement among the clinical experts for all three measurements: the intraclass correlation estimates were between 0.78 and 0.86; the variance between the experts was found to be small for both the calcaneal tilt and cuboid height, with values of 2.4 and 3.6, respectively; and the proportion of variance that the experts contributed was small (< 10%) for all measurements (Table 2).

**Table 2:** Inter-observer analysis of manual measurements of calcaneal tilt, cuboid height and Meary's angle taken by five clinical experts (ICC2 = intraclass correlation coefficient 2; CI = confidence interval; IOV = inter-observer variance; IIV = inter-image variance; POV = proportion of variance explained by observer differences).

Measurement	ICC2	ICC2 95% CI	IOV	IIV	POV
**Calcaneal tilt**	0.85	[0.78–0.90]	2.4	38.6	5%
**Cuboid height**	0.78	[0.67–0.85]	3.6	32.0	9%
**Meary's angle**	0.86	[0.81–0.90]	6.6	168.3	3%

**Fig. 5:**
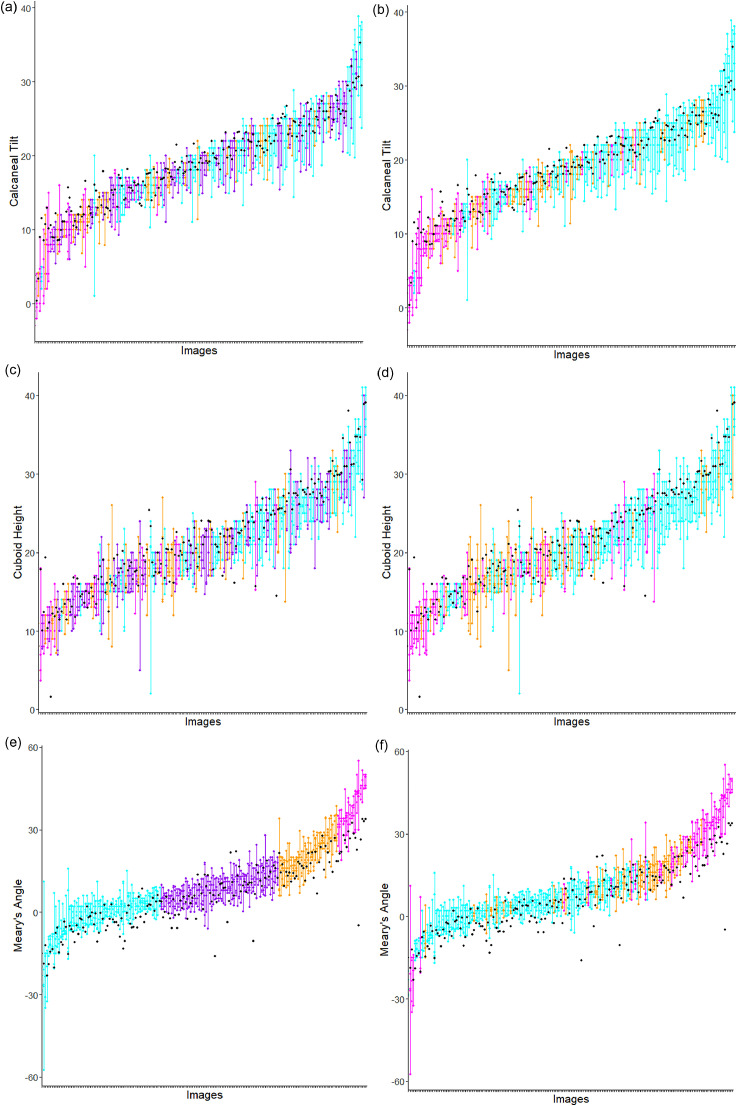
CT (a), CH (c) and MA (e) are manual measurement values per severity class based on Meary's angle: normal (cyan), mild (purple), moderate (orange), severe (magenta). CT (b), CH (d) and MA (f) are manual measurement values per severity class based on clinical review: normal (cyan), mild (orange), severe (magenta). All five manual measurements per image are shown and joined with a line for better visibility. Automatic results (black) are largely in agreement with manual measurements (i.e. black dots are within spread of manual measurements per image). The images in all plots are sorted by the mean over the respective manual measurements.

For all 200 images, the Meary's angle based severity classification resulted in 71 normal, 70 mild, 40 moderate and 19 severe cases (images); although based on a recent clinical review of the data only 22 of the mild and 38 of the moderate cases definitely had foot collapse. Based on clinical review, the 200 images included 108 normal, 45 mild and 47 severe cases (images). The data collected did not allow to discriminate between congenital and acquired foot collapse. However, the vast majority of the patients in the Trust's podiatry clinic are diabetics attending with symptoms of Charcot arthropathy. Thus, it is assumed that most of our cases of foot collapse were acquired, rather than congenital. For the 188 images included in the geometric analyses, the Meary's angle based severity classification resulted in 69 normal, 68 mild, 34 moderate and 17 severe cases (images), and the clinical review based classification resulted in 106 normal, 41 mild and 41 severe cases. Fig. 5 illustrates the spread of the manual measurements per image included in the geometric analyses, highlighting the severity classifications.

### 3.3 Point-finder

The point-finder results were obtained based on all 200 images using 10-fold cross-validation; each fold included a balanced number of images (*n* = 20) and subjects (*n* = 7 or *n* = 8).

**Fig. 6:**
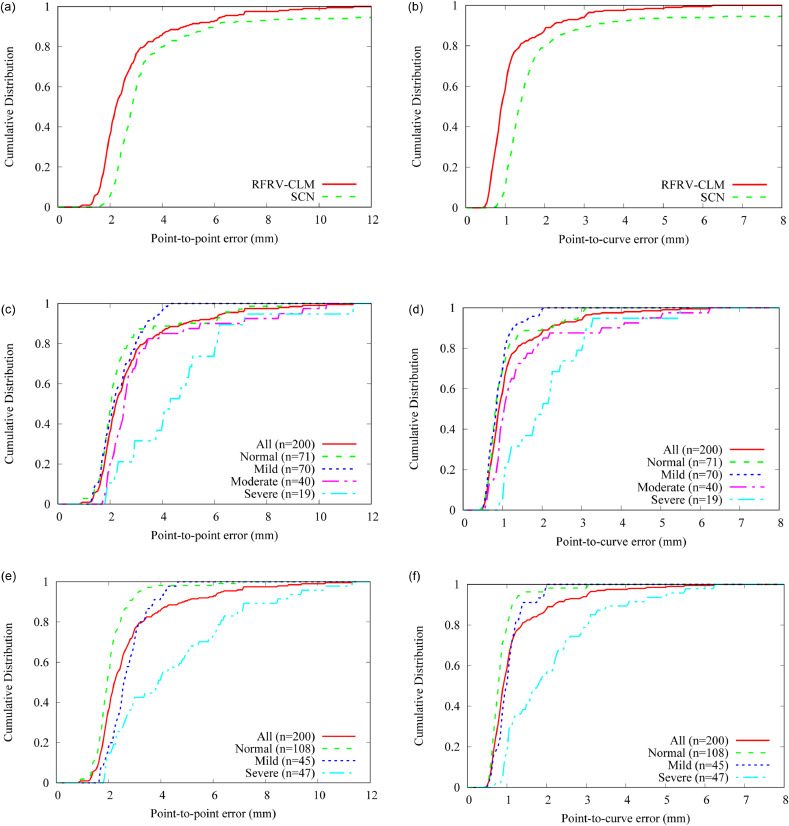
Fully automatic point-finder performance for locating the 61 points as in Fig. 4 in all 200 images: (a, b) showing the performance for both the RFRV-CLM [15] and the SCN [16] methods; all results were obtained using the same data and 10-fold cross-validation experiments. (c, d) showing the RFRV-CLM performance for different severity classes with severity defined based on the average manual Meary's angle. (e, f) showing the RFRV-CLM performance for different severity classes with severity defined based on clinical review. The severity grouping was applied retrospectively to the point-finder results; the point positions of all groups were obtained using the same 10-fold cross-validation point-finder systems.

The cumulative density functions (CDFs) of the point-to-point error results for both the RFRV-CLM and the SCN methods show that the RFRV-CLM method yields more accurate and robust results (Fig. 6a–b). We used the RFRV-CLM method to provide the fully automatic point-finder results for this study. The point-finder system achieved a median point-to-point error of 2.2 mm, and a point-to-point error of less than 3 mm for 75% of all 200 images, and a median point-to-curve error of 0.9 mm, and a point-to-curve error of less than 1.3 mm for 75% of all 200 images.

The performance of the point-finder system appears to strongly depend on the severity of disease (defined based on Meary's angle or clinical review), with a markedly poorer performance for severe cases (Fig. 6c–f). For normal cases based on clinical review, the point-finder system achieved a median point-to-point error of 2.0 mm, and a point-to-point error of less than 3 mm for 92% of all 200 images, and a median point-to-curve error of 0.8 mm, and a point-to-curve error of less than 1.3 mm for 94% of all 200 images.

The developed RFRV-CLM point-finder system is freely available for non-commercial purposes from www.bone-finder.com.

### 3.4 Geometry-calculator

For all three measurements, there was no significant difference between the manual geometric measurements and the derived geometric measurements calculated using the point-finder results at a significance level of 0.05 for non-severe cases and at a significance level of 0.01 for all cases (Table 3). This suggests that the fully automatic system provides an unbiased estimate for these measurements and is in agreement with the observers. Fig. 5 shows the automatic results in context of the spread of the manual measurements per image. There was a strong correlation between the manual and the automatically derived measurements (PCC=0.71, PCC=0.84 and PCC=0.82 for calcaneal tilt, cuboid height and Meary's angle, respectively). Calcaneal tilt and cuboid height achieved a high percentage within range (78.2% and 83.0%, respectively). For Meary's angle, however, fewer derived measurements (58.0%) were within the range of manual measurements. Analysing the percentage within observer range for Meary's angle based on severity groups shows that this is lowest for severe cases (17.6% and 46.3% for severity based on Meary's angle and clinical review, respectively).

**Table 3:** Performance measures (bias, correlation, and within range agreement) for the comparison between (i) the manual measurements and the derived measurements based on automatically obtained point positions; and (ii) the manual measurements and the derived measurements based on the manual ground truth point positions (LMM = linear mixed-effects model; PCC = Pearson correlation coefficient; CI = confidence interval).

**Me**asurement	LMM		PCC (bootstrap	% within
	Bias estimate	*p*-value	95% CI)	observer range
*Calculations based on automatically obtained point positions*				
**Calcaneal tilt**	0.85	0.29	0.71 [0.66–0.76]	78.2
**Cuboid height**	1.08	0.28	0.84 [0.80–0.88]	83.0
**Meary's angle**	−3.54	0.03	0.82 [0.74–0.87]	58.0

*Calculations based on manual ground truth point positions*				
**Calcaneal tilt**	0.59	0.45	0.73 [0.68–0.77]	84.6
**Cuboid height**	1.29	0.21	0.87 [0.84–0.90]	81.4
**Meary's angle**	−1.85	0.19	0.89 [0.86–0.91]	65.4

*Calculations based on automatically obtained point positions*				
*when excluding severe cases based on clinical review*				
**Calcaneal tilt**	0.66	0.46	0.63 [0.56–0.69]	80.3
**Cuboid height**	1.29	0.24	0.86 [0.82–0.89]	86.4
**Meary's angle**	−2.59	0.09	0.89 [0.85–0.92]	61.2

Similar results (i.e. a high percentage within range of manual measurements for calcaneal tilt and cuboid height and only a moderate percentage for Meary's angle) were obtained when analysing the difference between the manual geometric measurements and the derived geometric measurements based on the *manual* ground truth point annotations (Table 3). In addition, there was a high agreement between the geometric measurements based on the automatically obtained point positions and the geometric measurements based on the manual ground truth point positions for all three measurements (PCC=0.95, PCC=0.92 and PCC=0.88 for calcaneal tilt, cuboid height and Meary's angle, respectively).

## 4 Discussion

We have developed a fully automatic software system to assess foot collapse on lateral weight-bearing foot radiographs, automatically calculating calcaneal tilt, cuboid height and Meary's angle. Our statistical analysis showed no significant difference between the automatically derived and manually taken measurements, suggesting that the system provides an unbiased estimate for these measurements and is in agreement with clinical experts. Further, we observed a similar result pattern for the derived measurements based on *both* the automatic point annotations and the manual point annotations. This implies that the point-finder results are sufficiently accurate and robust to automatically calculate calcaneal tilt, cuboid height and Meary's angle.

A high proportion of calcaneal tilt and cuboid height automatically derived measurements were within observer range but a lower percentage was apparent for Meary's angle. The moderate percentage of within range measurements indicates that the definition for the points-based calculation of the angle would benefit from further refinement. Difficulties in fitting Meary's angle in the context of the Charcot foot has been previously reported in the literature. In [3], this difficulty is attributed to the concomitance of talar head deformity in some advanced midfoot presentations, which may obscure visualisation of anatomy [1]. We have preliminary results (not published) which suggest that the definition of how to fit the metatarsal axis has a significant impact on how well the derived Meary's angle correlates with manual measurements. It may therefore be necessary to gain a better understanding of whether this may vary implicitly depending on foot collapse severity. The high agreement between the Meary's angle measurements based on the automatically obtained point positions versus based on the manual ground truth point positions further supports that the moderate performance for automatically calculating Meary's angle is not rooted in the performance of the point-finder but in the definition for the points-based calculation of Meary's angle.

A potential limitation of Meary's angle is illustrated in Fig. 4b. Our dataset contained three cases where the 1st metatarsal subluxed superiorly but maintained a relatively normal orientation. In these cases, Meary's angle can be almost normal, even in the presence of advanced midfoot collapse.

Fig. 6c–d show superior point-finder performance of some mild cases compared to normal cases based on severity defined on the average manual Meary's angle. However, when defining severity based on clinical review Fig. 6e–f show a clear pattern in degrading performance of the point-finder with disease progression. This difference in point-finder results depending on the definition of severity suggests that classifying cases based on Meary's angle may not accurately reflect the state of disease. This is also supported by the results of our clinical review of the data with respect to the presence of foot collapse which demonstrated limited agreement with the Meary's angle-based severity classification. This is likely because the classification does not consider any clinical symptoms, and because an increased Meary's angle does not necessarily relate to foot collapse but may be dependant upon positioning.

The expected application of such a system would be primarily in early diagnosing mild Charcot neuroarthropathy cases and monitoring the progression of moderate Charcot neuroarthropathy cases. In mild/early cases, accuracy is of high importance as small changes can be used to gauge treatment effect and highlight possible occult ligament failure. Moderate cases, defined by a Meary's angle 15° ≤ 30°, have been previously reported as clinically significant. Meary's angles > 27° have been linked to plantar ulceration [9], with other work finding that Meary's angles > 25° are linked to greater likelihood of amputation [17]. Thus, in moderate cases, alignment measures may be used to gauge prognosis and inform clinical decision making. Cases classed as severe may represent individuals of sufficiently advanced destructive change and clinical urgency for which obtaining an accurate Meary's angle provides little clinical utility. The presence of overt and advanced bony destruction will likely trigger a surgical consult. Further research into extreme ranges of angular alignment in the Charcot foot is warranted to confirm this.

This study used retrospectively collected data. One could argue that a prospective study design would have allowed for a standardised x-ray acquisition technique to reduce variabilities caused by image acquisition. However, in clinical practice a standardised x-ray acquisition technique is rarely applied or may be healthcare provider-specific. We used a retrospective study design to develop a system that is able to handle the variability in *routinely collected* clinical data.

Manually taking radiographic measurements is time-consuming and prone to inconsistencies. Automating radiographic assessment will save time, and has the potential to ensure equality of service across different hospitals. No data was collected regarding the time taken to manual acquire the discussed measurements. However, a similar study found an average of 667 s to take the required measurements to assess flatfoot [18]. An automated system will be able to generate the discussed measurements in less than 1 min and without human interaction.

Little research has been published in the area of automating radiographic measures for lateral weight-bearing foot radiographs. There is some work on automating measurements for assessing flatfoot [18], [19], [20]. Traditional image analysis methods were used in [19] and a convolutional neural network in [18] to automatically calculate the arch angle. In [20], a Random Forest-based method was used to automatically calculate four measurements including calcaneal tilt and Meary's angle. The method was assessed using intra-observer measurements for 48 images (not including any moderate or severe cases based on Meary's angle), yielding similar results to ours for calcaneal tilt but inferior correlation results for Meary's angle (no inter-observer variability was assessed). All of these studies appear to have primarily included cases of congenital flatfoot whereas our cohort almost exclusively included patients with acquired flatfoot. Whilst the loss of the longitudinal arch is a feature of both congenital and acquired flatfoot, the latter are often radiologically more challenging. This is due to the associated joint destruction and bony fragmentation which limits accurate delineation of the bones. To the best of our knowledge, no results have yet been reported on fully automatically measuring cuboid height.

Even though 200 images were included for the development of the system, this study is limited by the small number of subjects (79). We attribute the reduced point-finder performance for severe cases to the fact that it is the group with the largest variation in deformities but the fewest data available for training (19 images from 8 subjects). The performance of the system was validated using cross-validation experiments for both the point-finder and geometry-calculator to assess the generalizability of the system to unseen data. However, the software will need to be further validated on external data to better understand its real-world performance.

Future work will include increasing the number of images/subjects (using additional datasets) as well as the refinement of the points-based calculation of Meary's angle (e.g. metatarsal axis fitting). There are also potential applications of such an automated system outside of midfoot collapse, for example, for the automatic calculation of Bohler's angle in the context of calcaneal fractures. We consider this study to be a pilot study, conducted as an important and necessary first step in exploring the feasibility and potential effectiveness of such a system.

## Author contribution statement

All authors have made substantial contributions to data acquisition or study execution, manuscript preparation, and final approval of the submitted article. C. Lindner takes responsibility for the integrity of the work as a whole.

## Declaration of Competing Interest

The authors declare not to have any conflict of interest.
